# A264 ORAL IRON SUPPLEMENTATION SHAPES THE RECOVERY OF GUT THE MICROBIOTA AND PROMOTES CARCINOGENESIS IN THE APCMIN/+ MOUSE MODEL

**DOI:** 10.1093/jcag/gwac036.264

**Published:** 2023-03-07

**Authors:** T Cuisiniere, M Oliero, R Hajjar, A Calvé, G Fragoso, M M Santos

**Affiliations:** 1 Nutrition and Microbiome Laboratory - Institut du cancer de Montréal - Centre de recherche du Centre hospitalier de l’Université de Montréal (CRCHUM); 2 Department of Medicine, Faculty of Medicine - Université de Montréal, Montréal, Canada

## Abstract

**Background:**

Colorectal cancer (CRC) induces anemia in a large proportion of patients and is usually treated with oral iron supplementation. Surgery, the main treatment for CRC, is routinely accompanied by prophylactic antibiotics to avoid infection. Moreover, the concomitance of oral iron supplementation and antibiotics treatment is highly prevalent in general population. However, the combined effect of antibiotics and high luminal iron concentration in the gut on the microbiota and intestinal homeostasis remains unknown.

**Purpose:**

This study sought to identify possible deleterious effects of the recovery from antibiotics exposure of the gut microbiota under oral iron supplementation.

**Method:**

In order to assess the effects of luminal iron on the recovery of the gut microbiota, WT mice were subjected to antibiotic treatment with different concentrations of dietary iron. Then, to explore potential deleterious effects of this setup on CRC promotion and progression mediated by the human CRC gut microbiota, CRC model *Apc*^Min/+^ mice were subjected to antibiotics prior to gavage with fecal samples from anemic CRC patients, then fed different concentrations of dietary iron. The composition of the gut microbiota and its recovery after these interventions were assessed in stool samples by 16S rRNA. Gut microbiota functions were inferred by using the PICRUSt2, and short chain fatty acid (SCFA) concentration in feces were assessed by liquid-chromatography mass spectrometry. CRC progression and promotion were assessed by tumour counts and immunohistochemistry.

**Result(s):**

Recovery from antibiotics under high luminal iron concentration shifted the gut microbiota toward a Bacteroidetes phylum-dominant composition. Three bacterial species characterized as CRC initiators were significantly more abundant. Nitrogen, pentose phosphate, cyanoamino acid metabolisms were higher after recovery under oral iron supplementation. Antibiotic exposure induced a long-term increase in SCFAs linked to gut inflammation, propionate and succinate. For mice under high luminal iron concentration, they showed a lack of recovery in baseline levels of butyrate, a SCFA that inhibits cancer cell proliferation in the gut. In addition, an increase in CRC burden and progression were found in *Apc*^Min/+^ mice receiving fecal sample from patients and oral iron supplementation.

**Conclusion(s):**

Gut microbiota recovery from antibiotic exposure under oral iron supplementation is frequent in CRC patients, but is also common in the general population. This study identifies possible deleterious effects of the concomitance of these two disruptive agents of the gut microbiota on the CRC progression.

**Please acknowledge all funding agencies by checking the applicable boxes below:**

Other

**Please indicate your source of funding;:**

NSERC, ICM

**Disclosure of Interest:**

None Declared

